# Association between mineral-vitamin bolus supplementation during the breeding period and reproductive outcomes in dairy cows

**DOI:** 10.3389/fvets.2026.2004069

**Published:** 2026-10-30

**Authors:** Kamilė Kačiulytė, Saulius Urbonas, Robertas Stoškus, Šarūnė Sorkytė, Gintarė Vaičiulienė, Neringa Sutkevičienė, Toma Vilkonienė, Audronė Rekešiūtė

**Affiliations:** 1 Large Animal Clinic, Faculty of Veterinary Medicine, Lithuanian University of Health Sciences, Kaunas, Lithuania; 2 Animal Reproduction Laboratory, Faculty of Veterinary Medicine, Lithuanian University of Health Sciences, Kaunas, Lithuania

**Keywords:** artificial insemination, conception, dairy cows, magnesium, mineral-vitamin bolus, pregnancy, reproductive outcomes, reproductive performance

## Abstract

The period between calving and first artificial insemination (AI) is characterised by major metabolic adaptations that may influence subsequent reproductive performance in dairy cows. In this retrospective matched cohort study, we examined the association between mineral-vitamin bolus supplementation during the early breeding period and reproductive performance and blood biochemical parameters under commercial dairy farm conditions. Clinically healthy dairy cows were enrolled in matched pairs based on calving date and lactation number. Within each pair, one cow received the bolus, whereas the other served as a control. Allocation was not randomised. A total of 88 cows were included in the final analysis (45 supplemented and 43 control cows). Cows received two boluses at 51.13 ± 3.15 days in milk (DIM) and two additional boluses 14 days later. Each two-bolus dose provided a declared daily delivery of 2.4 g magnesium, 130 mg zinc, 36 mg copper, 1.5 mg selenium, 132,000 international units (IU) of vitamin A, 500 IU vitamin E, and 300 mg *β*-carotene over a 10-day release period. Reproductive outcomes included conception at first AI, pregnancy by 150 and 300 DIM, time to confirmed pregnancy, days to first oestrus, and days to first AI. Blood samples were collected at enrolment and 14 and 21 days later. After adjustment for baseline metabolic status, parity, and DIM at enrolment, bolus supplementation was associated with higher odds of conception at first AI (odds ratio (OR) = 3.45; 95% confidence interval (CI): 1.07–11.11; *p* = 0.038) and, as an exploratory endpoint, pregnancy by 300 DIM (OR = 3.80; 95% CI: 1.19–12.19; *p* = 0.025). No differences were observed in pregnancy by 150 DIM or time to confirmed pregnancy. Supplementation was not associated with the resumption of oestrus overall. An earlier resumption in multiparous cows was an exploratory subgroup finding. Supplemented cows had higher serum magnesium concentrations and lower lactate dehydrogenase (LDH) activity than controls (supplementation-by-time interaction, *p* < 0.001 and *p* = 0.025, respectively). Bolus supplementation was therefore associated with higher odds of conception and pregnancy and with altered mineral status. The difference in cumulative pregnancy rates emerged only after 180 DIM, more than 100 days after supplementation ended.

## 1 Introduction

Following parturition, high-producing dairy cows enter one of the most metabolically demanding phases of their production cycle. During early lactation, the abrupt increase in nutrient requirements for milk synthesis frequently exceeds dietary energy intake, resulting in negative energy balance (NEB) (1–3). This physiological adaptation is characterised by profound alterations in metabolic and endocrine function, including reduced circulating insulin and insulin-like growth factor-1 (IGF-1) concentrations, increased mobilisation of non-esterified fatty acids (NEFA), and changes in hepatic gluconeogenic capacity (4, 5). NEB also affects mineral homeostasis, and subclinical deficiencies of magnesium, phosphorus, calcium, and trace elements are widely reported in dairy herds (6–8). These metabolic and mineral imbalances may impair the recovery of normal hypothalamic–pituitary-ovarian function after calving, delay the resumption of ovarian cyclicity, and compromise reproductive performance (9, 10).

Reproductive efficiency is a major determinant of biological and economic sustainability in dairy production systems. Parameters such as the interval to first oestrus, interval to first insemination, conception rate, and pregnancy rate directly influence lifetime productivity and overall farm profitability (11–13). Economic assessments conducted in Europe have estimated that reproductive disorders may impose costs of approximately €100 per cow annually through reduced fertility, increased veterinary interventions, and involuntary culling (14). Despite advances in genetics, nutrition, and herd management, reproductive performance in high-yielding dairy cows remains suboptimal in many commercial production systems. Conception rates at first insemination nevertheless remain close to 40% in high-producing herds (15). The period between calving and the first insemination is widely recognised as a critical window during which metabolic and nutritional interventions may significantly influence subsequent reproductive outcomes (16, 17).

Beyond the resumption of ovarian cyclicity, successful conception and maintenance of pregnancy are critical determinants of reproductive efficiency. Nutritional and metabolic disturbances during the breeding period may adversely affect fertilisation and pregnancy maintenance. This may reduce the overall reproductive success (18, 19). Minerals and micronutrients play essential roles in the physiological processes underlying reproductive success (20). In dairy cows, magnesium deficiency may compromise reproductive performance by impairing follicular development and oestrus cyclicity, either alone or in combination with deficiencies of other minerals (21). Phosphorus is another essential nutrient involved in reproductive physiology through its central role in adenosine triphosphate (ATP) energy transfer and intracellular signalling (8). Inadequate phosphorus status during early lactation has been linked to delayed ovarian activity and reduced conception rates in dairy cows (22). Trace elements such as zinc, copper, and selenium, together with fat-soluble vitamins, contribute to reproductive performance through antioxidant, immune, and endocrine mechanisms. Deficiencies of these nutrients have been associated with impaired fertility and increased susceptibility to postpartum disorders (16, 23, 24).

To address these nutritional challenges, sustained-release mineral-vitamin boluses have been increasingly adopted as a practical strategy for targeted supplementation in commercial dairy herds. Administered orally, these boluses gradually dissolve within the reticulorumen, providing a continuous supply of minerals and vitamins over a period of days to weeks (25). Compared with short-term injectable preparations or conventional dietary supplementation, this delivery system may achieve a more sustained supply of nutrients and help maintain more stable circulating mineral concentrations (26). Consequently, mineral boluses have attracted growing interest as a management tool to support metabolic adaptation and reproductive performance during the postpartum and breeding periods. Parenteral supplementation has been examined more extensively. Repeated multimineral and multivitamin injections across the transition period improved antioxidant status and immune function in cows and their calves (27) and enhanced mammary immune function and colostrum quality in crossbred cows (28). In Holstein cows, injectable mineral supplementation around calving reduced uterine disease and hypocalcaemia, although reproductive indices did not differ between treatments (29). These studies concern parenteral delivery during the transition period rather than oral supplementation given during the breeding period itself. However, despite the biological rationale supporting their use, evidence regarding the association between mineral-vitamin bolus supplementation and reproductive performance remains limited. Existing studies have largely focused on mineral status, milk yield, or health outcomes during the transition period, whereas data evaluating fertility outcomes following supplementation during the early breeding period are scarce and inconsistent (30). Moreover, evidence on the relationship between sustained-release mineral supplementation during the breeding period and conception and pregnancy outcomes under commercial dairy farm conditions remains scarce. Therefore, further research is warranted to determine whether improvements in mineral status achieved through targeted supplementation can translate into measurable reproductive benefits. We hypothesised that mineral-vitamin bolus supplementation during the early breeding period would be associated with higher odds of conception and pregnancy, together with changes in mineral status and selected biochemical indicators.

Accordingly, the aim of this study was to evaluate the association between mineral-vitamin bolus supplementation during the early breeding period and reproductive performance and blood biochemical dynamics in dairy cows managed under commercial farm conditions. The specific objectives were: (i) to determine the association between bolus supplementation and conception at first artificial insemination (AI) and pregnancy by 150 and 300 days in milk (DIM); (ii) to assess associations between bolus supplementation and reproductive timing, including days to first oestrus and first artificial insemination; and (iii) to investigate changes in selected blood biochemical parameters following bolus administration in supplemented and control cows.

## 2 Materials and methods

### 2.1 Farm and herd management

The study was conducted on a commercial family-owned dairy farm located in Kazlų Rūda municipality, Lithuania. The farm maintained approximately 2,400 Holstein × Lithuanian Black-and-White cattle, including 1,100 lactating cows with an average annual milk yield of about 11,000 kg per cow. The average parity of lactating cows in the herd was 2.9. Cows were loose-housed in free-stall barns with individual cubicles. The barns were equipped with automated ventilation and temperature-activated fans.

Cows were fed a total mixed ration (TMR) designed for high-producing dairy cows and prepared using a self-propelled mixer wagon. The ration was based on grass and maize silage with a commercial compound concentrate; its full composition and the analysed nutrient and mineral content of the forages are presented in Supplementary Table S1. Forages were analysed by near-infrared spectroscopy and supplied approximately 35 g of magnesium and 312 g of potassium per cow daily, resulting in a potassium-to-magnesium ratio of approximately 9:1. Forage copper and zinc concentrations were 5.5 and 23 mg/kg dry matter, respectively. The mineral content of concentrates and premixes was not analysed; therefore, the reported values represent forage-derived mineral intake rather than total dietary supply. Cows had ad libitum access to commercial mineral salt as part of routine herd management and to clean drinking water. Fresh TMR was offered once daily, pushed up several times throughout the day, and refusals were removed daily. The same ration was fed to all study groups throughout the study, and feeding management remained unchanged.

The Herd Navigator system, integrated with DeLaval DelPro software, continuously collected and analysed data during milking. Cows were milked twice daily in a DeLaval side-by-side milking parlour with 2 × 30 stalls. Milk progesterone concentrations were monitored automatically from 45 DIM onwards using the Herd Navigator system (DeLaval, Sweden). Oestrus detection was primarily based on changes in milk progesterone concentrations over time. Cows displaying visual signs of oestrus before a Herd Navigator alert were also included in the breeding programme. The decision to inseminate was made following a clinical reproductive examination performed by a veterinarian. A voluntary waiting period of 60 DIM was applied, after which cows were inseminated according to oestrus alerts generated by the system. Between 90 and 100 DIM, cows with persistently low milk progesterone concentrations indicative of absent luteal activity were treated with 100 μg gonadorelin (Ovarelin, Ceva Sante Animale, France). This management protocol was routinely applied to all cows regardless of study group. Study cows calved between February and May.

### 2.2 Animals, study design and bolus administration

This study is a retrospective analysis of data generated during an on-farm evaluation of a commercially available dietetic mineral-vitamin bolus. The evaluation was initiated and conducted by the farm as part of routine herd management: farm staff selected the cows, administered the boluses, and arranged the blood sampling. The investigators had no role in its conduct and obtained the herd records, laboratory results, and reproductive outcomes only after the observation period had ended, at which point the research question and analysis plan were defined. The design is therefore a matched cohort study analysed retrospectively, and allocation to supplementation was not randomised. A total of 100 cows (50 matched pairs) were enrolled. Twelve cows were excluded after enrolment: eight because one or more of the three scheduled blood samples were missing, two because of clinical disease during follow-up, one because it died, and one because it was culled. Reasons for exclusion by group are shown in Figure 1. Excluded and retained cows are compared in Supplementary Table S2. Consequently, data from 88 cows were included in the final analysis, comprising 45 supplemented cows and 43 controls. This included 39 complete enrolment pairs and 10 cows whose matched enrolment partner had been excluded. Because exclusions during follow-up resulted in incomplete matched pairs, the primary analyses were conducted at the individual-cow level, with the pairing retained in a sensitivity analysis restricted to the complete pairs (Supplementary Table S3). All selected cows belonged to the same lactating-cow group and were maintained under identical housing, feeding, and management conditions throughout the study period.

**Figure 1:**
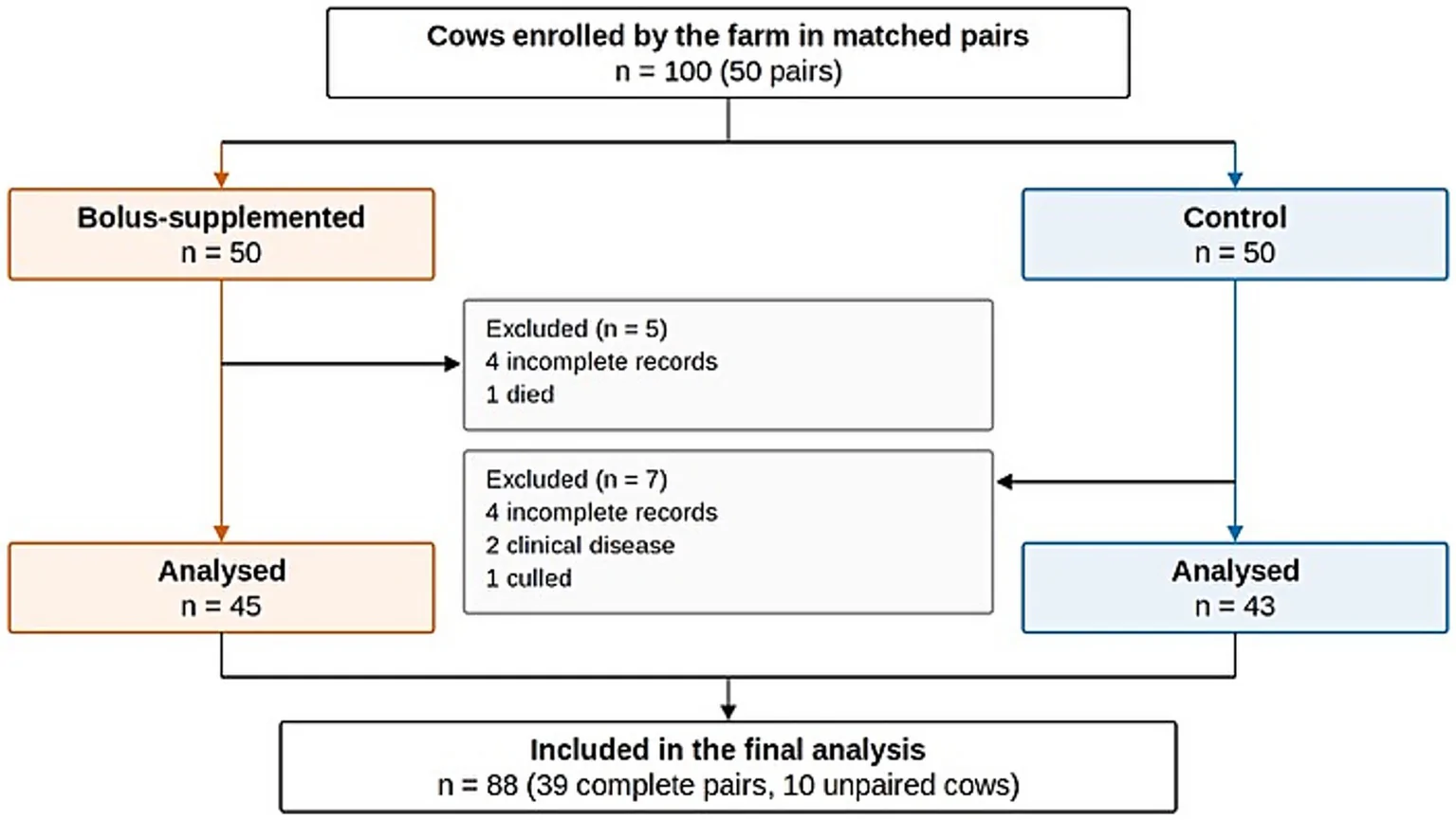
Flow of cows through the study. One hundred cows were enrolled by the farm in 50 matched pairs based on calving date and lactation number, and within each pair one cow received the bolus. Allocation was not randomised. Exclusions after enrolment are shown separately for each group. The final analysis included 88 cows, comprising 39 complete pairs and 10 cows whose pair member had been excluded.

Eligible cows were assessed at approximately 50 DIM before the first bolus administration. Inclusion criteria were clinical health, uncomplicated calving, and parity between one and five lactations. The study population comprised 30 primiparous and 58 multiparous cows. Baseline health status was determined from veterinary records and the herd-management programme, including previous diagnoses of uterine disease, mastitis, and metabolic disorders. Body condition score (BCS) was assessed by a single veterinarian using a 1–5 scale at enrolment in both groups. Baseline milk yield was defined as the average daily milk yield accumulated from calving until enrolment (36.5 ± 10.9 kg/day).

Cows were enrolled in pairs on the same day by farm staff. Cows designated to receive the bolus were selected by the herd veterinarian in the order in which they calved, rather than on the basis of body condition, health status, metabolic indicators, or previous reproductive performance; a control cow was then matched to each of them from cows of the same or an adjacent lactation number with a similar calving date and daily milk yield. Allocation was thus systematic rather than randomised, and selection of supplemented cows was independent of their individual health and production characteristics. No selection took place within an already formed pair. The supplemented cow was identified first, and the pair was then built around her. Matching was close, with paired cows differing by a median of 1 day in calving date (interquartile range (IQR) 0–1), by no more than one lactation, and by a median of 5.3 kg/day in daily milk yield at enrolment (IQR 2.2–9.0). Allocation preceded the baseline blood sample, which was taken immediately before the first bolus was given, and all serum analyses were carried out after the sampling period had ended; serum results were therefore unavailable at the time of allocation. Milk fat, milk protein, and milk *β*-hydroxybutyrate were recorded continuously by the herd management system and were in principle visible to farm staff, but were not used as selection criteria.

Cows assigned to the supplementation group received a commercially available mineral-vitamin bolus registered as a dietetic mineral feed (Bolutech™ Refresh; Neolait S.A.S., France), marketed as a nutritional supplement intended by the manufacturer to support oestrus expression and reproductive performance. Each bolus weighed 65 g. According to the manufacturer, the recommended dose of two boluses provides a sustained release of trace elements and vitamins over an average period of 10 days, corresponding to a declared daily delivery of 130 mg zinc, 36 mg copper, 1.5 mg selenium, 132,000 IU of vitamin A, 500 IU vitamin E, and 300 mg *β*-carotene. The detailed nutrient composition of the bolus is presented in Table 1.

**Table 1:** Analytical composition and additive content of the mineral-vitamin bolus used in the study (per bolus).

Component category	Component	Content
Analytical composition	Phosphorus	6.2%
	Magnesium	18.8%
	Calcium	0.5%
	Sodium	2.9%
Additives		
Trace elements	E6 zinc	650 mg
	E4 copper	180 mg
	3b814 selenium	7.5 mg
Vitamins	3a672a vitamin A	660,000 IU
	3a700 vitamin E	2,500 IU
	3a160 *β*-carotene	1,500 mg
Main mineral sources		Magnesium oxide	Included
	Magnesium phosphate	Included
	Monosodium phosphate	Included
	Magnesium stearate	Included

IU, international units.

Boluses were administered orally. Two boluses were given at 51.13 ± 3.15 DIM (range 44–57 DIM), followed by two additional boluses 14 days later. The first administration was timed approximately 10 days before the end of the 60-day voluntary waiting period, in accordance with the manufacturer’s recommendation for use during the breeding period. For this study, the early breeding period corresponded to the period around the end of the voluntary waiting period and the initiation of breeding activities.

The primary outcome was conception at first AI. Secondary outcomes included pregnancy by 150 DIM, pregnancy by 300 DIM, time to pregnancy, days to first oestrus, days to first insemination, and serum biochemical variables.

### 2.3 Blood sampling, laboratory analyses and data collection

Blood samples were collected at enrolment (d0), immediately before bolus administration, and 14 and 21 days later. Samples were obtained from the *coccygeal vein* into clot-activator tubes and centrifuged at 3,000 × g for 5 min, and the serum was stored at −20 °C until analysis. Serum concentrations of phosphorus, magnesium, calcium, sodium, albumin, and total protein, as well as lactate dehydrogenase (LDH) activity, were determined using validated colourimetric assays on an automated biochemical analyser (SELECTRA Junior; Vital Scientific, The Netherlands) with commercial reagents (Spinreact, Spain). Samples were visually inspected for haemolysis before analysis.

Milk *β*-hydroxybutyrate (mBHB), milk fat, and milk protein data were obtained from the DeLaval DelPro herd management system. Milk *β*-hydroxybutyrate concentrations were used as an indicator of postpartum ketotic status. The ketosis-risk score generated by the herd management system was not included in the statistical analyses because it was derived from mBHB and milk composition variables.

Oestrus was detected using the Herd Navigator system based on milk progesterone concentration profiles and automated alerts, as described above. First oestrus was defined as the first oestrus event recorded in the herd management software, identified either by a Herd Navigator progesterone alert from 45 DIM onwards or, before 45 DIM, by visual signs recorded by farm staff during routine daily management. Oestrus events recorded before 45 DIM therefore rest on visual observation alone. Artificial insemination was performed after 60 DIM according to the farm reproductive management protocol. No synchronisation protocols were applied during the study period. Pregnancy was initially diagnosed based on milk progesterone concentration changes approximately 20 days after AI and was subsequently reconfirmed by ultrasonography (iScan mini, Draminski, Poland) at 60 days after AI. Only ultrasonographically confirmed pregnancies were counted as outcome events, and cows not confirmed at 60 days were classified as non-pregnant following that insemination regardless of the earlier progesterone profile. Conception at first AI was defined as a pregnancy confirmed by ultrasonography 60 days after the first insemination. Cows re-inseminated before that confirmation were not counted as having conceived to the first service. Farm insemination records were reviewed to verify conception outcomes. Cows diagnosed as non-pregnant returned to the breeding programme and were re-inseminated at the next detected oestrus.

### 2.4 Ethical approval

All procedures involving animals were conducted as part of routine herd management and preceded the retrospective study. Ethical approval for the retrospective analysis of herd records and biological samples was granted by the Bioethics Centre of the Lithuanian University of Health Sciences (approval No. 2026-BEC2-0897, May 2026). Written informed consent was obtained from the herd owner for the use of farm records and biological data.

### 2.5 Statistical analysis

This study is reported in accordance with the Strengthening the Reporting of Observational Studies in Epidemiology (STROBE) statement. Analyses were performed in IBM SPSS Statistics (version 30.0; IBM Corp., Armonk, NY, USA), except for Firth penalised logistic regression and survival analyses, which were performed in R version 4.6.1 (logistf 1.26.1; survival 3.8.11). Normality was assessed using the Shapiro–Wilk test. Continuous variables are presented as mean ± standard deviation (SD), and categorical variables as frequencies (%). No *a priori* sample-size calculation was performed, as the study population was determined by the number of eligible cows available. Interpretation is therefore based on the magnitude and precision of the effect estimates rather than on dichotomous significance testing.

Baseline characteristics and d0 serum values were compared using independent-samples *t*-tests, the Mann–Whitney *U* test for non-normally distributed variables, and Pearson’s chi-squared test without continuity correction. Lactate dehydrogenase was log-transformed and is reported as back-transformed geometric means with between-group ratios and 95% confidence intervals. Covariates were selected *a priori* based on biological relevance.

Blood biochemical parameters across the three sampling points were analysed using linear mixed models with group, time, and their interaction as fixed effects, cow as the subject, and an unstructured covariance matrix; denominator degrees of freedom were approximated using the Satterthwaite method. Conception at first AI and pregnancy by 150 and 300 DIM were analysed using multivariable logistic regression adjusted for baseline mBHB, milk fat, milk protein, parity, and DIM at enrolment, and the analyses were repeated using Firth penalised likelihood because outcome events were few relative to the number of predictors. Days to first oestrus and first insemination were analysed using general linear models with bolus supplementation, parity, and their interaction as fixed effects; both events were recorded in all cows. Time to confirmed pregnancy was analysed using Kaplan–Meier estimates with the log-rank test and Cox regression adjusted for parity, censoring cows not confirmed pregnant at 300 DIM; the proportional hazards assumption was assessed using scaled Schoenfeld residuals. Inseminations received and services per conception were compared using the Mann–Whitney U test.

Conception at first AI was designated *a priori* as the primary outcome and evaluated at *p* < 0.05 without correction; all other endpoints were secondary or exploratory and interpreted as hypothesis-generating. Results with *p*-values between 0.05 and 0.10 are reported descriptively and interpreted cautiously.

Three sensitivity analyses were performed. First, because the outcome events (25 for conception at first AI, 64 for pregnancy by 300 DIM) gave only about four per covariate, a reduced model containing supplementation, parity, and DIM at enrolment was fitted in addition to the fully adjusted model, whose covariates were prespecified on biological grounds and kept unchanged across outcomes for comparability (Supplementary Table S4). Second, conditional logistic regression stratified on the enrolment pair was fitted to the 39 pairs in which both members completed follow-up (*n* = 78), with a pair-stratified Cox model for time to confirmed pregnancy (Supplementary Table S3). Third, gonadorelin treatment was added as a covariate to the conception and pregnancy models.

## 3 Results

### 3.1 Baseline characteristics

Bolus-supplemented cows had higher baseline milk fat (mean difference 0.27%; 95% confidence interval (CI): 0.08–0.45; *p* = 0.005) and milk protein (0.13%; 95% CI: 0.04–0.23; *p* = 0.004), and lower milk *β*-hydroxybutyrate (−0.005 mmol/L; 95% CI: −0.009 to −0.001; *p* = 0.028) than controls, although the median was 0.060 mmol/L in both groups and all values fell between 0.04 and 0.08 mmol/L. The milk fat-to-protein ratio, a composite indicator of early-lactation energy balance, did not differ between groups (1.33 in supplemented cows and 1.30 in control cows; mean difference 0.03; 95% CI: −0.02 to 0.07; *p* = 0.302). The groups did not differ in DIM at enrolment, average daily milk yield, body condition score, prior disease, parity, or the distribution of calving months (all *p* ≥ 0.23; Table 2).

**Table 2:** Baseline characteristics of the study groups.

Variable	Control (*n* = 43)	Supplemented (*n* = 45)	*p*
DIM at enrolment (mean ± SD)	51.30 ± 2.66	51.13 ± 3.15	0.787
Average daily milk yield at enrolment, kg/day (mean ± SD)	36.47 ± 11.13	36.48 ± 10.74	0.998
Body condition score, median (IQR)	3.00 (2.75–3.00)	3.00 (2.75–3.00)	0.840
Milk fat, % (mean ± SD)	4.71 ± 0.46	4.98 ± 0.42	0.005
Milk protein, % (mean ± SD)	3.62 ± 0.21	3.76 ± 0.22	0.004
Milk fat-to-protein ratio (mean ± SD)	1.30 ± 0.12	1.33 ± 0.11	0.302
Baseline milk BHB, mmol/L, median (IQR)	0.060 (0.050–0.065)	0.060 (0.050–0.060)	0.028
Disease before enrolment, *n* (%)	20 (46.5)	25 (55.6)	0.396
Parity, *n* (%)			0.23
Primiparous	12 (27.9)	18 (40.0)	
Multiparous	31 (72.1)	27 (60.0)	

Independent-samples *t*-test for DIM, milk yield, milk fat, and milk protein; Mann–Whitney *U* test for body condition score and milk *β*-hydroxybutyrate; Pearson chi-squared test without continuity correction for categorical variables. BHB, *β*-hydroxybutyrate; IQR, interquartile range; DIM, days in milk; SD, standard deviation.

Cows excluded after enrolment (*n* = 12) did not differ from those retained in parity, DIM, milk composition, or milk *β*-hydroxybutyrate, but had lower milk yield at enrolment (28.0 ± 7.0 versus 37.6 ± 7.3 kg/day, *p* = 0.001; Supplementary Table S2).

At baseline, serum concentrations of phosphorus, magnesium, calcium, sodium, albumin, and total protein were comparable between groups (all *p* > 0.05). Baseline lactate dehydrogenase activity was numerically higher in control cows than in bolus-supplemented cows, but this difference was not statistically significant (ratio 1.23, 95% CI: 0.89–1.69; *p* = 0.207). The three variables that differed at baseline were included as covariates in all reproductive models.

### 3.2 Reproductive outcomes

The proportion of cows conceiving at the first AI was higher in the bolus-supplemented group (37.8%) than in the control group (18.6%), and this difference was statistically significant in the unadjusted analysis (*χ*^2^(1) = 3.97, *p* = 0.046). In the adjusted logistic regression model, bolus supplementation remained associated with higher odds of conception at first artificial insemination (odds ratio (OR) = 3.45; 95% CI: 1.07–11.11; *p* = 0.038). The Firth-penalised estimate for the same model was comparable (OR = 3.08; 95% CI: 1.06–9.93; *p* = 0.038). In the reduced model containing only supplementation, parity, and DIM at enrolment, the estimate was attenuated and no longer reached significance (OR = 2.48; 95% CI: 0.92–6.67; *p* = 0.073). Across all specifications, it ranged from 2.4 to 3.5 (Supplementary Table S4). Baseline mBHB concentration was not significantly associated with conception at first insemination (OR = 1.67 per 0.01 mmol/L; 95% CI: 0.96–2.90; *p* = 0.070). No other covariates included in the model were significantly associated with conceiving at first AI.

The proportion of cows pregnant by 150 DIM did not differ between groups (57.8%, *n* = 26 versus 55.8%, *n* = 24; *χ*^2^(1) = 0.03, *p* = 0.853; adjusted OR = 1.20; 95% CI: 0.44–3.29; *p* = 0.718; Firth penalised likelihood: OR = 1.19; 95% CI: 0.45–3.15; *p* = 0.727). Multiparous cows were less likely than primiparous cows to be pregnant by this point (OR = 0.34; 95% CI: 0.12–0.92; *p* = 0.033).

The proportion of cows confirmed pregnant by 300 DIM was higher in bolus-supplemented cows (82.2%) than in controls (62.8%), and this difference was statistically significant in the unadjusted analysis (*χ*^2^(1) = 4.19, *p* = 0.041). In the adjusted logistic regression model, bolus supplementation was associated with higher odds of pregnancy (OR = 3.80; 95% CI: 1.19–12.19; *p* = 0.025), and Firth penalised likelihood estimation gave comparable results (OR = 3.34; 95% CI: 1.15–10.62; *p* = 0.027). Baseline mBHB, milk fat, milk protein, parity, and DIM at enrolment were not independently associated with pregnancy by 300 DIM (all *p* > 0.10) (Table 3). Because the groups were comparable at 150 DIM and separated only later in the breeding period (Figure 2), this endpoint is treated as exploratory.

**Table 3:** Multivariable logistic regression models for conception at first artificial insemination and pregnancy by 300 DIM.

Predictor	Conception at 1st AI OR (95% CI)	*p*	Pregnancy by 300 DIM OR (95% CI)	*p*
Bolus supplementation	3.45 (1.07–11.11)	0.038	3.80 (1.19–12.19)	0.025
Baseline milk BHB (per 0.01 mmol/L)	1.67 (0.96–2.90)	0.070	1.20 (0.68–2.10)	0.535
Milk fat (%)	0.62 (0.17–2.24)	0.463	0.43 (0.12–1.62)	0.211
Milk protein (%)	3.18 (0.23–43.1)	0.385	1.14 (0.09–14.1)	0.919
DIM at enrolment	1.00 (0.84–1.18)	0.973	1.16 (0.97–1.39)	0.105
Parity (primiparous vs. multiparous)	0.49 (0.18–1.34)	0.162	0.43 (0.13–1.38)	0.154

Both models were adjusted for all predictors shown. Firth penalised likelihood estimation gave comparable estimates for bolus supplementation (conception at first AI: OR 3.08, 95% CI 1.06–9.93, *p* = 0.038; pregnancy by 300 DIM: OR 3.34, 95% CI 1.15–10.62, *p* = 0.027). AI, artificial insemination; BHB, *β*-hydroxybutyrate; CI, confidence interval; DIM, days in milk; OR, odds ratio.

**Figure 2:**
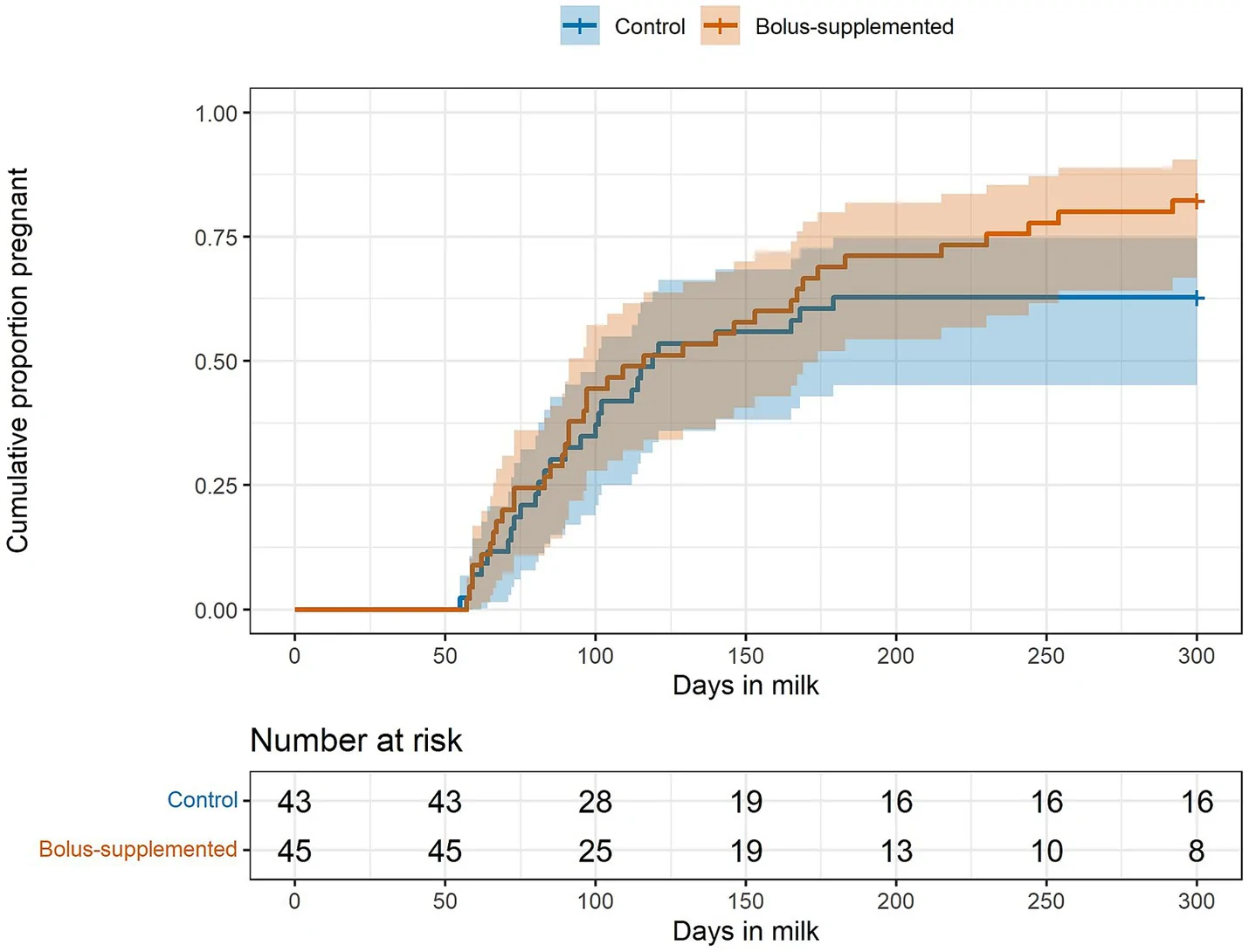
Kaplan–Meier curves for the cumulative proportion of cows confirmed pregnant, by group. Cows not confirmed pregnant were censored at 300 days in milk (DIM). Shaded areas are 95% confidence intervals, and the table below the curves shows the number of cows remaining at risk at each time point. Log-rank *p* = 0.167.

Median time from calving to confirmed pregnancy was 116 days (95% CI: 91–174) in supplemented cows and 119 days (95% CI: 100–not reached) in controls (log-rank *χ*^2^(1) = 1.91, *p* = 0.167). In the Cox model adjusted for parity, supplementation was not associated with the rate of pregnancy (hazard ratio (HR) = 1.29; 95% CI: 0.77–2.15; *p* = 0.330). Multiparous cows conceived more slowly than primiparous cows (HR = 0.62; 95% CI: 0.37–1.05; *p* = 0.074). The proportional hazards assumption was satisfied for both covariates and for the whole model (global *p* = 0.29). Kaplan–Meier curves are shown in Figure 2.

In the pair-stratified sensitivity analysis (39 pairs, *n* = 78), the estimate for conception at first AI remained in the same direction but was less precise (OR = 2.50; 95% CI: 0.78–7.97), as did the estimate for pregnancy by 300 DIM, and no between-group differences were detected for the remaining outcomes in either analysis (Supplementary Table S3). Precision was expected to fall, because outcome-concordant pairs are uninformative in the conditional likelihood and the 10 unpaired cows were excluded.

First oestrus was observed between 41 and 156 DIM. After adjustment for parity, bolus supplementation was not associated with days to first oestrus overall (adjusted mean difference −2.1 days; 95% CI: −12.2 to 8.0; *F*(1, 84) = 0.17, *p* = 0.678). Parity alone was not associated with this outcome (*F*(1, 84) = 0.01, *p* = 0.940). However, a significant bolus × parity interaction was observed (difference between parity groups 22.3 days; 95% CI: 0.2–44.4; *F*(1, 84) = 4.22, *p* = 0.043). Multiparous supplemented cows reached first detected oestrus 13.6 days earlier than multiparous control cows (95% CI: 1.0–26.3; *p* = 0.035), whereas among primiparous cows the difference was in the opposite direction and was not significant (9.0 days later in supplemented cows; 95% CI: −8.9 to 26.9; *p* = 0.319). The primiparous subgroup comprised 30 cows (18 supplemented cows, 12 control cows). This interaction and the subgroup contrasts derived from it were exploratory and were not adjusted for multiple comparisons.

First insemination was recorded between 49 and 156 DIM. After adjustment for parity, the estimated mean interval to first insemination was 87.1 days (95% CI: 79.1–95.0) in control cows and 80.8 days (95% CI: 73.6–87.9) in supplemented cows. Bolus supplementation was not associated with days to first insemination (adjusted mean difference −6.3 days; 95% CI: −17.0 to 4.4; *F*(1, 84) = 1.38, *p* = 0.244). Neither parity (*p* = 0.590) nor the bolus × parity interaction (*p* = 0.160) was significantly associated with this outcome.

Breeding exposure was comparable between groups. The total number of inseminations per cow did not differ between supplemented and control cows (median 2, IQR 1–4 versus median 2, IQR 2–3; *U* = 898.0, *p* = 0.552). Among cows that conceived, the number of services per conception was similar between groups (median 2 in both groups; *U* = 485.5, *p* = 0.842).

Prolonged anoestrus managed with gonadorelin according to the herd protocol was recorded in 9 supplemented and 9 control cows (20.0% versus 20.9%; *χ*^2^(1) = 0.01, *p* = 0.914). Adding gonadorelin treatment to the adjusted models did not materially change the estimates for conception at first AI (OR = 3.47; 95% CI: 1.07–11.22; *p* = 0.038), pregnancy by 300 DIM (OR = 3.79; 95% CI: 1.14–12.66; *p* = 0.030), or the time to confirmed pregnancy (HR = 1.26; 95% CI: 0.76–2.08; *p* = 0.366).

### 3.3 Serum biochemical variables

Serum magnesium concentrations changed differently over time in bolus-supplemented and control cows [group × time interaction: *F*(2, 86.0) = 11.05, *p* < 0.001]. Magnesium concentrations increased over the study period in supplemented cows but decreased in controls (Figure 3). Averaged across all sampling points, supplemented cows had higher serum magnesium concentrations than controls (mean difference 0.048 mmol/L, 95% CI: 0.022–0.075; *p* < 0.001). No between-group difference was detected at baseline (*p* = 0.381), whereas higher concentrations were observed in supplemented cows at both d14 (*p* = 0.024) and d21 (*p* < 0.001) after Bonferroni adjustment. Parity was not associated with serum magnesium concentration (*p* = 0.292) (Table 4).

**Figure 3:**
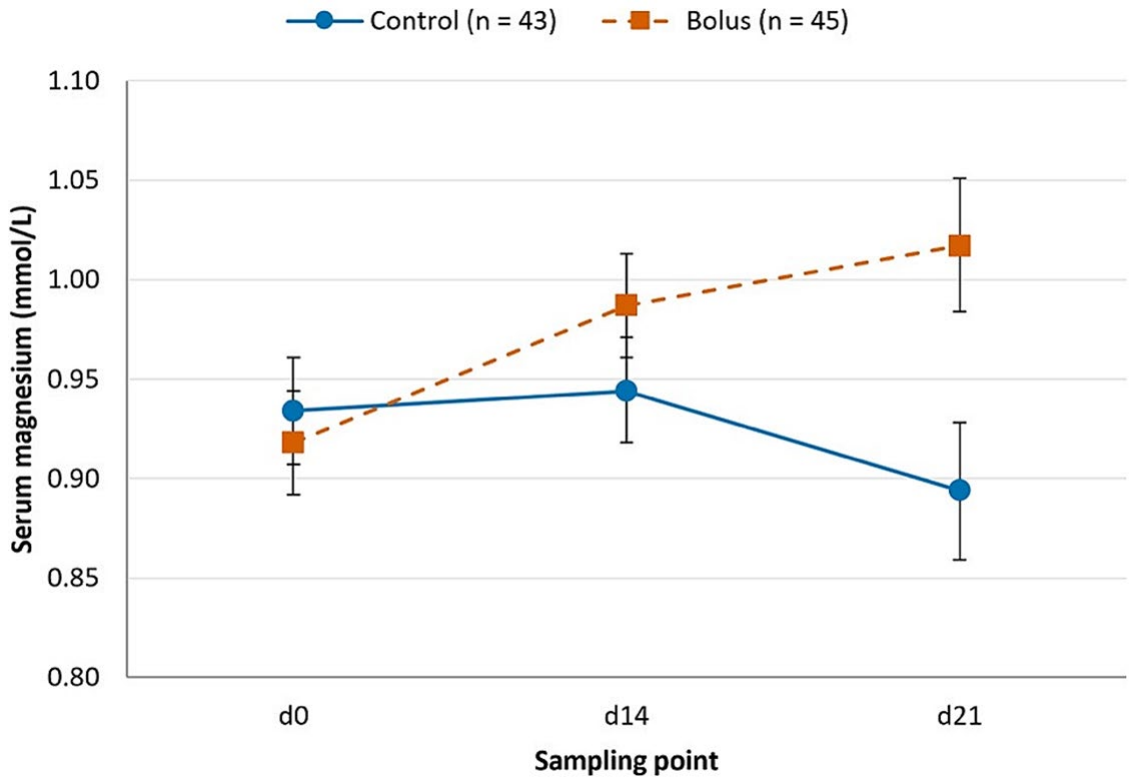
Serum magnesium concentration (mmol/L) in control (*n* = 43) and bolus-supplemented (*n* = 45) cows at enrolment (d0) and 14 and 21 days later. Points are estimated marginal means from the linear mixed model, and error bars are 95% confidence intervals. Group × time interaction: *p* < 0.001.

**Table 4:** Serum magnesium and lactate dehydrogenase (LDH) by group and sampling point.

Parameter	Group	d0	d14	d21	*F* (2, 86.0)	*p* (group × time)	*p* (parity)
Magnesium (mmol/L)	Control	0.93 (0.014)	0.94 (0.014)	0.89 (0.017)	11.05	**<0.001**	0.292
	Bolus	0.92 (0.013)	0.99 (0.013)	1.02 (0.017)			
LDH (U/L, geometric mean)	Control	1,506 (1,186–1,914)	1,490 (1,134–1,955)	1886 (1,412–2,515)	3.83	**0.025**	0.487
	Bolus	1,212 (968–1,518)	1,104 (852–1,430)	972 (738–1,279)			

Values are estimated marginal means (standard errors) from linear mixed models with an unstructured covariance matrix. LDH values are back-transformed geometric means (95% confidence intervals). Denominator degrees of freedom were approximated using the Satterthwaite method and ranged from 85.0 to 86.0 across model terms. The remaining analytes are presented in Supplementary Table S5. Bold *p*-values indicate a statistically significant group × time interaction (*p* < 0.05).

d0 = before bolus administration; d14 = 14 days later; d21 = 21 days later.

Serum LDH activity also showed a significant group × time interaction [*F*(2, 86.0) = 3.83, *p* = 0.025]. LDH activity increased over time in control cows but decreased in bolus-supplemented cows (Figure 4). No significant between-group differences were observed at baseline or d14; however, LDH activity was significantly higher in control cows than in supplemented cows at d21 (ratio 1.94, 95% CI: 1.31–2.87; *p* = 0.001). Parity was not associated with LDH activity (*p* = 0.487).

**Figure 4:**
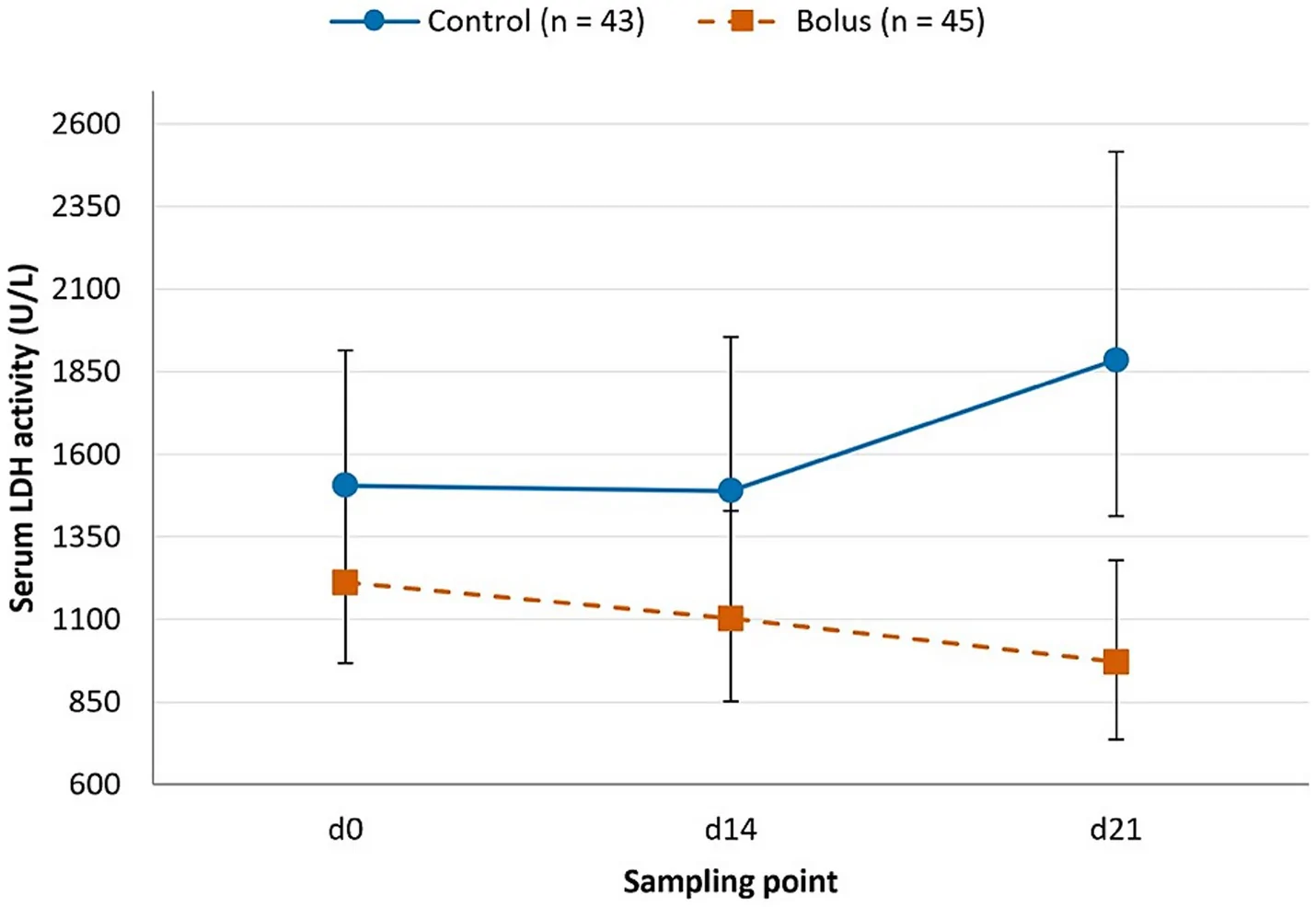
Serum lactate dehydrogenase activity (U/L) in control (*n* = 43) and bolus-supplemented (*n* = 45) cows at enrolment (d0) and 14 and 21 days later. Points are back-transformed geometric means from the linear mixed model, and error bars are 95% confidence intervals. Group × time interaction: *p* = 0.025.

Serum phosphorus, calcium, sodium, albumin, and total protein showed no significant group × time interaction (all *p* ≥ 0.137) and no overall association with bolus supplementation. Estimated marginal means at each sampling point are presented in Supplementary Table S5. Parity effects were evident independently of supplementation: phosphorus (2.06 versus 1.93 mmol/L; *p* < 0.001) and calcium (2.44 versus 2.40 mmol/L; *p* < 0.001) were higher in primiparous cows, whereas total protein was higher in multiparous cows (76.6 versus 72.7 g/L; *p* < 0.001). Parity was not associated with sodium (*p* = 0.52) or albumin (*p* = 0.50).

## 4 Discussion

Under commercial conditions, cows receiving a mineral-vitamin bolus during the early breeding period had significantly higher odds of pregnancy than unsupplemented animals, despite showing no overall difference in the timing of first oestrus or insemination. Supplemented cows also had higher serum magnesium concentrations and lower lactate dehydrogenase activity. These biochemical differences indicate that supplementation altered mineral status, but they cannot establish which, if any, of the bolus constituents accounted for the reproductive associations.

Although conception at first AI was already higher in supplemented cows, a greater cumulative pregnancy proportion could in principle arise if supplemented cows simply received more breeding opportunities. This does not appear to apply: all cows were followed until confirmed pregnancy or 300 DIM, so breeding opportunity was equal by design, and neither the number of inseminations received nor the number of services per conception differed between groups. The timing of the difference is also informative. At 150 DIM, the two groups were almost identical, and the advantage in supplemented cows emerged only over the remainder of the breeding period; consistent with this, the time to confirmed pregnancy did not differ between groups. The association was therefore with the probability of eventually establishing pregnancy rather than with the rate at which it was achieved. The number of days to first oestrus reflects how quickly ovarian activity resumes after calving, whereas pregnancy depends on the entire sequence of fertilisation, embryo survival, and uterine support across the breeding period (18, 31). A supplement could therefore be associated with pregnancy without changing the timing of first oestrus.

Early lactation places heavy demands on mineral and vitamin reserves. Negative energy balance in this period disrupts mineral homeostasis and endocrine signalling, impairing follicular development and the uterine environment on which successful conception depends (22, 32, 33). A sustained supply of minerals and fat-soluble vitamins across the breeding window may buffer these disturbances, supporting the metabolic conditions required not merely to conceive but to maintain an established pregnancy (34–36). Comparable associations between trace-element status and reproductive performance have been reported in dairy cattle (37), though heterogeneity in supplementation strategy and study design limits direct comparison (16, 31).

The hypothesis was partially supported: both conception at first AI and pregnancy by 300 DIM were higher in supplemented cows, whereas overall reproductive timing was unaffected. The one exception was a parity-dependent difference in the resumption of oestrus, with multiparous supplemented cows returning to oestrus approximately 2 weeks earlier than multiparous controls and no such difference in primiparous animals. The greater metabolic demand of multiparous cows in early lactation could explain this difference in response, but given the small primiparous subgroup, the absence of adjustment for multiple comparisons, and the exploratory status of this endpoint, the observation requires confirmation.

Pregnancy by 300 DIM was chosen as the cumulative endpoint because cows not confirmed pregnant by this point effectively complete the lactation open and are usually culled, making it the reproductive outcome of greatest economic relevance at the herd level. The difference in cumulative pregnancy nevertheless emerged mainly after 180 DIM, more than 100 days after supplementation ended. A direct effect of the bolus at that stage is biologically implausible, and the pattern may reflect chance in a small sample or unmeasured differences between groups. This endpoint is therefore treated as exploratory.

Baseline mBHB showed a non-significant positive association with conception at first insemination, a direction opposite to the reduced fertility consistently reported with elevated ketone concentrations in early lactation (38, 39). Concentrations in this cohort spanned a narrow range at or below the thresholds used to identify hyperketonaemia in milk (40), so the estimate was derived across an essentially normal span of values. The association appears statistically unstable, and we therefore draw no biological conclusion from it.

Magnesium moved in opposite directions in the two groups: it rose in supplemented cows and fell in controls, so that by the final sampling point supplemented cows had serum magnesium concentrations that were 0.124 mmol/L higher. The decline in controls fits the higher magnesium demand and often marginal magnesium status of cows in early lactation (7, 20). Each administration of two boluses supplied approximately 24 g of magnesium, or about 2.4 g daily over the declared 10-day release period – roughly 7% of the magnesium provided by the forage portion of the ration. Although modest, this increment was delivered against a forage potassium concentration of about 20 g/kg dry matter and a potassium-to-magnesium ratio of approximately 9:1. High dietary potassium is a recognised inhibitor of ruminal magnesium absorption (41). Because serum magnesium in ruminants is not under tight endocrine control and depends mainly on absorption and renal excretion (7), a small but sustained increase in supply can measurably shift circulating concentrations. Magnesium is not thought to act on fertility directly, but it is required by many enzymes involved in energy metabolism and hormone synthesis, and magnesium status has been related to reproductive performance in dairy cattle (21, 42). Metabolic status more broadly is a recognised determinant of reproductive outcome in early lactation (2, 43). The increase in serum magnesium therefore provides evidence that supplementation altered mineral status. However, the present study cannot determine whether magnesium itself mediated the reproductive associations.

The observed associations cannot be attributed to magnesium alone. The bolus also supplied zinc, copper, and selenium as glycine chelates, which are less susceptible to ruminal antagonism than inorganic sources (26). These nutrients, together with vitamin E and *β*-carotene, participate in antioxidant defence and immune regulation and may influence fertilisation and early embryo survival (17, 23, 37). Forage copper and zinc were below the reference values applied by the analysing laboratory, but these values describe the forage only. The mineral content of the compound concentrate and premix was not determined, and all cows had free access to commercial mineral salt, so total dietary supply could not be quantified and no conclusion about mineral adequacy can be drawn. Because circulating trace element and vitamin concentrations were not measured, any contribution of these nutrients remains hypothetical.

LDH activity rose in control cows over the sampling period and declined in supplemented cows. LDH is a cytosolic enzyme released into circulation following cellular damage or metabolic stress, and elevated serum activity has been associated with tissue turnover and metabolic strain during the demanding early-lactation period (43, 44). The rise observed in control cows between the second and third sampling points is therefore consistent with the metabolic pressure typical of this stage. Earlier studies have reported similar changes in serum LDH in supplemented early-lactation cows and declining activity in metabolically stable animals (45, 46). Although lower LDH activity in supplemented cows may suggest reduced tissue turnover or metabolic strain, the biological relevance of this finding remains uncertain. LDH is a relatively non-specific biomarker and was not measured alongside complementary markers such as creatine kinase, aspartate aminotransferase, acute-phase proteins, or markers of oxidative stress.

Phosphorus, calcium, sodium, albumin, and total protein showed no significant group × time interaction. These analytes are under tight homeostatic control through endocrine, renal, and hepatic mechanisms (46–50) and would not be expected to respond to short-term mineral supplementation in clinically healthy cows; concentrations remained within normal ranges in both groups throughout.

Parity was significantly associated with phosphorus, calcium, and total protein concentrations. Phosphorus and calcium were higher in primiparous cows, in agreement with reports of parity-related differences in mineral concentrations (51), possibly reflecting the greater mineral demands of younger, still-growing animals. In contrast, total protein was higher in multiparous cows, which would follow from the progressive accumulation of immunoglobulins across successive lactations and greater cumulative antigenic exposure with age (51, 52). These parity-related differences, although independent of bolus treatment, match previous reports of parity-dependent variation in the metabolic and biochemical profile of dairy cows (52) and were accounted for by adjusting for parity in all models.

Several limitations should be considered, and together they mean that no causal relationship between supplementation and the reproductive outcomes can be established from these data. Cows were not randomly allocated, and although pair members calved within a median of 1 day of one another and were managed identically, the groups differed at baseline in milk fat, milk protein, and milk *β*-hydroxybutyrate. However, the fat-to-protein ratio was comparable and all milk *β*-hydroxybutyrate values were well below any threshold for subclinical ketosis. These were included as covariates in all reproductive models, but residual confounding cannot be excluded. Baseline lactate dehydrogenase activity was also numerically higher in control cows, so the LDH findings are interpreted with caution. No *a priori* power calculation was performed, and the moderate sample size is reflected in wide confidence intervals, although Firth penalised likelihood estimation yielded comparable effect estimates. Twelve cows (5 supplemented cows and 7 control cows) were excluded after enrolment, and attrition bias cannot be excluded. Eight of the twelve exclusions followed a missing blood sample and were balanced between groups. The remaining four, related to disease, death, or culling, occurred mostly in the control group, potentially biasing the results against rather than in favour of the association reported here. Exclusions also left 10 pairs with one remaining member and removed one pair entirely, so the analyses were conducted at the individual-cow level. In the matched-pair sensitivity analysis, the estimates were attenuated but remained in the same direction (Supplementary Table S3). In addition, the difference in cumulative pregnancy emerged only after 180 DIM, more than 100 days after supplementation ended, limiting direct attribution of this finding to the bolus intervention.

No hormonal, ovarian, or uterine measurements were available, and circulating trace element and vitamin concentrations were not determined, so the underlying mechanisms could not be examined directly. Mineral content was analysed for the forages only. Cows in both groups also had ad libitum access to commercial mineral salt, whose individual intake was not documented. Finally, the study was conducted on a single farm, and all cows calved between February and May, which constrains generalisability to other herds, breeds, management systems, and calving seasons. These findings should be regarded as hypothesis-generating and confirmed in adequately powered, randomised, multi-herd studies that include measurement of circulating trace element and vitamin status, ovarian activity, and reproductive hormone profiles.

## 5 Conclusion

In this retrospective observational study, mineral-vitamin bolus supplementation during the early breeding period was associated with higher first-service conception and cumulative pregnancy proportions in dairy cows. The observational design and the temporal pattern of the findings preclude causal inference. Conception at first AI occurred at a median of 69 DIM, within days of the second bolus, whereas the difference in cumulative pregnancy by 300 DIM emerged only after 180 DIM, more than 100 days after supplementation ended. The latter is therefore reported as an exploratory association that cannot be attributed directly to the supplementation. Supplemented cows also had higher serum magnesium concentrations and lower lactate dehydrogenase activity. The magnesium difference shows that supplementation altered mineral status. Lactate dehydrogenase is non-specific and was measured without complementary markers, so the lower activity cannot on its own indicate reduced metabolic stress. Bolus supplementation was not associated with days to first insemination, the proportion pregnant by 150 DIM, the time to confirmed pregnancy, or most measured biochemical variables. An earlier resumption of oestrus was observed only in multiparous supplemented cows. This was an exploratory subgroup finding and requires confirmation. Given the single-herd setting, the non-randomised allocation, and the moderate sample size, the results are best treated as hypothesis-generating. Randomised multi-herd studies with adequate power are needed to establish whether targeted mineral-vitamin supplementation during the breeding period improves fertility and to clarify the mechanisms involved.

## Data Availability

The original contributions presented in the study are included in the article/Supplementary material, further inquiries can be directed to the corresponding author.
