# Supplementary material for: Association between mineral-vitamin bolus supplementation during the breeding period and reproductive outcomes in dairy cows
Source: Front Vet Sci. 2026 Oct 30;13:2004069. doi: 10.3389/fvets.2026.2004069 (PMC13761333; doi:10.3389/fvets.2026.2004069)
Supplement: Supplementary file 1 [file Table_1.docx]

**Supplementary Table S1.** Composition of the total mixed ration and analysed nutrient and mineral content of the forages. Forages were analysed by near-infrared spectroscopy (Eurofins Agro, in cooperation with Pieno Tyrimai, Lithuania); the analyses correspond to the silage batches fed during the study period. The compound concentrate was based on cereals, protein meals, feed yeast, rumen-protected fat, and mineral premix; its mineral content was not analysed. Total ration values were calculated using the farm ration formulation software. DM = dry matter.

| **Item** | **Grass silage** | **Maize silage** | **Compound concentrate** | **Molasses** |
| --- | --- | --- | --- | --- |
| Amount fed, kg fresh weight/cow/day | 24.0 | 18.0 | 5.0 | 1.5 |
| Dry matter, g/kg | 412 | 320 | 886 | 750 |
| Contribution to ration dry matter, % | 48 | 28 | 21 | 3 |
| **Analysed composition of forages** | | | | |
| Crude protein, g/kg DM | 131 | 71 | – | – |
| Neutral detergent fibre, g/kg DM | 516 | 424 | – | – |
| Starch, g/kg DM | – | 269 | – | – |
| Net energy for lactation, MJ/kg DM | 5.6 | 6.8 | – | – |
| Magnesium, g/kg DM | 2.7 | 1.4 | – | – |
| Potassium, g/kg DM | 25.7 | 10.1 | – | – |
| Calcium, g/kg DM | 9.9 | 3.0 | – | – |
| Phosphorus, g/kg DM | 3.1 | 2.0 | – | – |
| Copper, mg/kg DM | 7.0 | 3.0 | – | – |
| Zinc, mg/kg DM | 27 | 16 | – | – |
| **Total ration** | | | | |
| Dry matter intake, kg/cow/day | 21.2 |  |  |  |
| Crude protein, % DM | 14.1 |  |  |  |
| Neutral detergent fibre, % DM | 39 |  |  |  |
| Starch, % DM | 13.5 |  |  |  |
| Sugars, % DM | 7.7 |  |  |  |
| Net energy for lactation, MJ/kg DM | 6.5 (1.55 Mcal/kg DM) |  |  |  |
| **Forage-derived mineral supply** | | | | |
| Magnesium supplied by forages, g/cow/day | 35 |  |  |  |
| Potassium supplied by forages, g/cow/day | 312 |  |  |  |
| Forage potassium-to-magnesium ratio | 9:1 |  |  |  |
