# Supplementary material for: Association between mineral-vitamin bolus supplementation during the breeding period and reproductive outcomes in dairy cows
Source: Front Vet Sci. 2026 Oct 30;13:2004069. doi: 10.3389/fvets.2026.2004069 (PMC13761333; doi:10.3389/fvets.2026.2004069)
Supplement: Supplementary file 2 [file Table_2.docx]

**Supplementary Table S2.** Comparison of cows excluded after enrolment with those retained in the analysis. Twelve of the 100 enrolled cows were excluded after enrolment, five supplemented and seven control cows. Exclusions by group and reason are shown in Figure 1. Eight followed a missing blood sample, were equally distributed between groups (p = 1.000) and were unrelated to any characteristic of the cow. The remaining four, related to clinical disease, death or culling, comprised one supplemented and three control cows (p = 0.617). The two cows excluded for clinical disease had acute febrile illness and clinical mastitis. All exclusions occurred after bolus administration.

| **Characteristic** | **Excluded (n = 12)** | **Retained (n = 88)** | **p** |
| --- | --- | --- | --- |
| Parity (lactation number) | 1.83 ± 1.03 | 2.11 ± 1.14 | 0.404 |
| Days in milk at enrolment | 51.5 ± 2.4 | 51.2 ± 2.9 | 0.962 |
| Milk yield at enrolment, kg/day | 28.0 ± 7.0 | 37.6 ± 7.3 | **0.001** |
| Milk fat, % | 4.73 ± 0.58 | 4.86 ± 0.47 | 0.410 |
| Milk protein, % | 3.67 ± 0.22 | 3.68 ± 0.23 | 0.728 |
| Milk β-hydroxybutyrate, mmol/L | 0.06 ± 0.01 | 0.06 ± 0.05 | 0.931 |
| Multiparous, n (%) | 6 (50.0) | 58 (65.9) | 0.342 |
| Allocated to bolus, n (%) | 5 (41.7) | 45 (51.1) | 0.760 |

*Values are mean ± SD unless stated otherwise. Continuous variables were compared with the Mann–Whitney U test and categorical variables with Fisher's exact test. Excluded cows had lower milk yield at enrolment, consistent with the subsequent development of disease or culling. One pair lost both members, leaving 39 complete pairs and 10 cows whose pair member had been excluded.*
