# Supplementary material for: Association between mineral-vitamin bolus supplementation during the breeding period and reproductive outcomes in dairy cows
Source: Front Vet Sci. 2026 Oct 30;13:2004069. doi: 10.3389/fvets.2026.2004069 (PMC13761333; doi:10.3389/fvets.2026.2004069)
Supplement: Supplementary file 3 [file Table_3.docx]

**Supplementary Table S3.** Sensitivity analyses preserving the original matched-pair design: conditional logistic regression stratified on the enrolment pair, restricted to the 39 pairs in which both members completed follow-up (n = 78 cows).

| **Outcome** | **Individual-cow model (n = 88)** | | **Matched-pair model (n = 78)** | | **Discordant pairs (bolus+ / control+)** |
| --- | --- | --- | --- | --- | --- |
|  | **Estimate (95% CI)** | **p** | **Estimate (95% CI)** | **p** |  |
| Conception at first AI | 3.45 (1.07–11.11) | 0.038 | 2.50 (0.78–7.97) | 0.121 | 14 (10 / 4) |
| Pregnancy by 300 DIM | 3.80 (1.19–12.19) | 0.025 | 1.83 (0.68–4.96) | 0.232 | 17 (11 / 6) |
| Pregnancy by 150 DIM | 1.20 (0.44–3.29) | 0.718 | 0.83 (0.36–1.93) | 0.670 | 22 (10 / 12) |
| Time to confirmed pregnancy | 1.29 (0.77–2.15) | 0.330 | 0.81 (0.43–1.53) | 0.517 | — |

*AI, artificial insemination; CI, confidence interval; DIM, days in milk. Estimates are odds ratios, except for time to confirmed pregnancy, which is a hazard ratio. Individual-cow models were adjusted for baseline milk β-hydroxybutyrate, milk fat, milk protein, parity and DIM at enrolment. Matched-pair models contain supplementation as the only covariate, because stratification controls all matching variables and all pair-level characteristics. Only outcome-discordant pairs contribute to the conditional likelihood, and “bolus+ / control+” gives the number of such pairs in which the supplemented and the control cow, respectively, achieved the outcome. Time to confirmed pregnancy was analysed with Cox models, adjusted for parity in the individual-cow analysis and stratified on pair in the matched analysis, censored at 300 DIM.*
