# Supplementary material for: Association between mineral-vitamin bolus supplementation during the breeding period and reproductive outcomes in dairy cows
Source: Front Vet Sci. 2026 Oct 30;13:2004069. doi: 10.3389/fvets.2026.2004069 (PMC13761333; doi:10.3389/fvets.2026.2004069)
Supplement: Supplementary file 4 [file Table_4.docx]

**Supplementary Table S4.** Sensitivity of the estimates for bolus supplementation to model specification (n = 88).

| **Model specification** | **Covariates** | **Conception at first AI OR (95 % CI)** | **Pregnancy by 300 DIM OR (95 % CI)** |
| --- | --- | --- | --- |
| Unadjusted | none | 2.66 (1.00–7.05) | 2.74 (1.03–7.32) |
| Reduced model | parity, DIM at enrolment | 2.48 (0.92–6.67) | 2.75 (0.99–7.63) |
| Reduced model, Firth | parity, DIM at enrolment | 2.36 (0.92–6.38) | 2.59 (0.99–7.21) |
| Fully adjusted (primary) | mBHB, fat, protein, parity, DIM at enrolment | 3.45 (1.07–11.11) | 3.80 (1.19–12.19) |
| Fully adjusted, Firth | mBHB, fat, protein, parity, DIM at enrolment | 3.08 (1.06–9.93) | 3.34 (1.15–10.62) |
| Fully adjusted + gonadorelin | as above plus gonadorelin | 3.47 (1.07–11.22) | 3.79 (1.14–12.66) |

*AI, artificial insemination; CI, confidence interval; DIM, days in milk; mBHB, milk β-hydroxybutyrate; OR, odds ratio. The fully adjusted model was specified a priori and is the model reported in the main text. Firth estimates use penalised likelihood. Estimates were consistent in direction across all specifications, but the confidence intervals were wide and included or approached unity in the reduced models, so the magnitude of the association is not precisely estimated.*
