# Supplementary material for: Association between mineral-vitamin bolus supplementation during the breeding period and reproductive outcomes in dairy cows
Source: Front Vet Sci. 2026 Oct 30;13:2004069. doi: 10.3389/fvets.2026.2004069 (PMC13761333; doi:10.3389/fvets.2026.2004069)
Supplement: Supplementary file 5 [file Table_5.docx]

**Supplementary Table S5.** Serum phosphorus, calcium, sodium, albumin and total protein by group and sampling point. Values are estimated marginal means (standard errors) from linear mixed models with bolus supplementation, time and their interaction as fixed effects, adjusted for parity, with cow as subject and an unstructured covariance matrix. d0 = at enrolment, d14 = 14 days, d21 = 21 days thereafter. Denominator degrees of freedom were approximated using the Satterthwaite method.

| **Parameter** | **Group** | **d0** | **d14** | **d21** | **F (2, 86.0)** | **p (group × time)** | **p (parity)** |
| --- | --- | --- | --- | --- | --- | --- | --- |
| Phosphorus (mmol/L) | Control | 1.99 (0.037) | 2.01 (0.037) | 1.97 (0.034) | 1.33 | 0.270 | <0.001 |
|  | Bolus | 1.98 (0.036) | 2.07 (0.035) | 1.94 (0.033) |  |  |  |
| Calcium (mmol/L) | Control | 2.39 (0.027) | 2.45 (0.016) | 2.41 (0.028) | 0.76 | 0.470 | <0.001 |
|  | Bolus | 2.44 (0.026) | 2.45 (0.015) | 2.41 (0.027) |  |  |  |
| Sodium (mmol/L) | Control | 139.5 (1.83) | 141.4 (1.00) | 136.8 (1.04) | 0.64 | 0.530 | 0.518 |
|  | Bolus | 141.3 (1.78) | 140.7 (0.96) | 135.8 (1.00) |  |  |  |
| Albumin (g/L) | Control | 35.5 (0.35) | 36.0 (0.23) | 36.0 (0.30) | 2.04 | 0.137 | 0.495 |
|  | Bolus | 35.6 (0.34) | 36.0 (0.22) | 35.4 (0.29) |  |  |  |
| Total protein (g/L) | Control | 73.5 (1.05) | 74.9 (0.90) | 74.2 (0.84) | 0.57 | 0.570 | <0.001 |
|  | Bolus | 75.3 (1.01) | 75.2 (0.86) | 74.7 (0.79) |  |  |  |
